# Prevalence of Noise-Induced Tinnitus in Adults Aged 15 to 25 Years: A Cross-Sectional Study

**DOI:** 10.7759/cureus.32081

**Published:** 2022-11-30

**Authors:** Aseel K Haji, Abdullah A Qashar, Shoog H Alqahtani, Roaa M Masarit, Tala S AlSindi, Elsaeid M Ali-Eldin

**Affiliations:** 1 College of Medicine and Surgery, Umm Al-Qura University, Makkah, SAU; 2 Otolaryngology - Head and Neck Surgery, King Abdullah Medical City, Makkah, SAU

**Keywords:** hearing loss, young adults, tinnitus, noise exposure, noise-induced tinnitus

## Abstract

Background

Tinnitus is a common complaint in the general population. Subjective tinnitus is defined as a conscious perception of sound with nonexistent external stimuli. Its exact pathophysiology remains unclear. Therefore, this study aimed to determine the prevalence of noise-induced tinnitus among adults aged 15-25 years in Makkah, Saudi Arabia.

Study design

Convenience sampling was used for participant recruitment using an online survey that was distributed online between February and April 2022. The participants performed audiometric hearing tests provided by the investigators. Hearing tests were performed at frequencies of 250, 500, 1000, 2000, 4000, and 8000 Hz. The test was considered normal if the achieved thresholds were 25 dB HL or less in at least four of the tested frequencies. Those with normal results were asked to fill out a survey inquiring about their demographic information, presence of tinnitus, and tinnitus functional index.

Results

We included 119 young adults aged 15-25 years. Regarding tinnitus prevalence, 27 (22.7%) adults reported the development of tinnitus after exposure to loud noise, 39 (32.8%) had tinnitus of unknown cause, and 53 (44.5%) had no tinnitus. Regarding the continuity of sound, it was continuous in 14.8% of noise-induced tinnitus, compared to 38.5% of the other group, with a statistical significance of (P=.037).

Conclusion

The current study revealed high prevalence of tinnitus, which was also suggested by the literature. Several triggers are purportedly related to the development of tinnitus. Constant exposure to loud noise is considered a significant risk factor for tinnitus. Young adults require proper education about the causes of tinnitus and other hearing abnormalities. More importantly, methods to protect and maintain their ear health.

## Introduction

Tinnitus is a common complaint in the general population; it is reportedly related to various conditions that are either subjective or objective [1]. Subjective tinnitus is described as the conscious perception of sounds with nonexistent external stimuli [2]. It can range from mild to severe, and temporary to chronic which disturbs and impacts the quality of life of affected individuals. It can interfere with sleep patterns, concentration, emotional status, and daily activities [3]. The exact pathophysiology remains incompletely understood; however, central or peripheral mechanisms have been suggested [4]. Central tinnitus begins in the brain due to the hyperactivity of nerve fibers in the auditory centers. Moreover, peripheral tinnitus may be mediated by malfunctioning of cochlear outer hair cells, consequently increasing cochlear activity [5,6]. Overexposure to noise, whether by listening to loud music or at festival events, has become a common occurrence, especially by young adults [7]. This constant overexposure is considered a significant risk factor for tinnitus [8]. Other risk factors include head injury, ototoxic medication, hearing loss, and depression [1]. Gilles et al. studied the prevalence of noise-induced tinnitus in high school students and found it to be as high as 74.9% in students with temporary tinnitus and 18.3% in those with permeant tinnitus [9]. Higher rates have been found among university students [7]. Detecting prevalence is the first substantial step in assessing the burden of a condition and in developing prevention strategies. Due to the lack of local data, this study aimed to determine the prevalence of noise-induced tinnitus among adults aged 15-25 in Makkah city, Saudi Arabia.

## Materials and methods

This cross-sectional study assesses the prevalence of self-reported noise-induced tinnitus among adults aged 15-25 years. This study was approved by the Biomedical Ethics Committee of Umm Al-Qura University (approval HAPO-02-K-012-2022-02-954).

Participants

The participants were recruited by convenience sampling using an online invitation, which was distributed on social media platforms (WhatsApp and Twitter) between February and April 2022. Individuals aged 15-25 years who were Makkah residents and willing to attend the clinic to undergo pure-tone audiometric hearing tests were included. Participants with a history of noise exposure, regardless of the cause and duration, were also included. Patients with abnormal audiometric hearing test results; previous positive diagnoses of hearing loss, vertigo, or Meniere’s disease; or a positive history of ototoxic drug intake were excluded. 

The sample size was calculated using the following formula (n=(Z^2 P(1-P))/d^2\). Where n represents the minimum required number, Z is the level of significance, P is the expected prevalence from the literature, and d represents the level of precision. The calculated minimum sample size was 76 individuals when the level of significance was set at 95%. The expected prevalence was 73% as reported by Degeest et al. [10], and 10% as level of precision.

Procedure

Two hundred and seventy-nine people accepted the invitation, and only 119 individuals approached the clinic for the pure-tone audiometric hearing test. Verbal consent was gained following the explanation of study aims and procedures. The test was conducted by an audiologist using headphones in sound-proof chambers. A cover was placed on the lower part of the glass window to conceal the examiner’s hand movements. Furthermore, tones were played at irregular intervals and durations to prevent pattern prediction by the examinee and to ensure accurate sound perception. The test aimed to measure hearing thresholds at frequencies of 250, 500, 1000, 2000, 4000, and 8000 Hz. The test was considered normal if the achieved thresholds were 25 dB HL or less in at least four of the tested frequencies [11]. Participants were then asked to complete a survey consisting of multiple sections. The first inquired about demographic data, followed by questions to evaluate the presence of tinnitus. Those with positive tinnitus were asked whether the development of tinnitus was following the exposure to loud noise or unknown. Subsequently they were categorized into two groups, labeled as noise-induced tinnitus group, and tinnitus of unknown cause group. They were then asked to complete the tinnitus functional index (TFI) in the final section. Data were collected using Google forms, transferred to Excel for cleaning, then to SPSS (IBM Corp., Armonk, NY, USA) for analysis.

Tinnitus Functional Index

The TFI was developed to assess the negative influence of tinnitus on eight specific domains. It includes the intrusiveness of tinnitus, control ability, cognition, sleep problems, auditory issues, relaxation ability, quality of life, and emotional distress. Data were collected using a validated Arabic version of the survey created by the University of Oregon Health and Science [12]. The total score of the TFI was determined by summing the points of the answered questions, dividing the sum by the number of questions, and multiplying it by 10. Each domain was calculated separately. According to the total score, the participants were categorized into three groups: scores below 25 were categorized as mild, those between 25 and 50 were considered to have significant impairment, and those more than 50 were severely impaired [12]. 

Data analysis

After data extraction, the data were cleaned, coded, and transferred to the statistical software IBM SPSS version 22. Two-tailed tests were used for all statistical analyses. Statistical significance was set at p < 0.05. Based on the frequency and percent distribution, descriptive analysis was performed for the adults’ biodemographics, type of tinnitus, noise exposure, noise-related data, and severity of tinnitus using the TFI scale. Cross-tabulation was performed to compare all groups regarding relevant bio-demographic data, tinnitus-related data, and tinnitus severity. The relationship significance was tested using the Pearson Chi-Square test as well as the exact probability tests due to the distributions of small frequencies.

## Results

A total of 175 individuals responded to the survey out of which 126 people consented and presented to the clinic for the test. Seven participants did not meet the inclusion criteria and were excluded.

**Figure 1:**
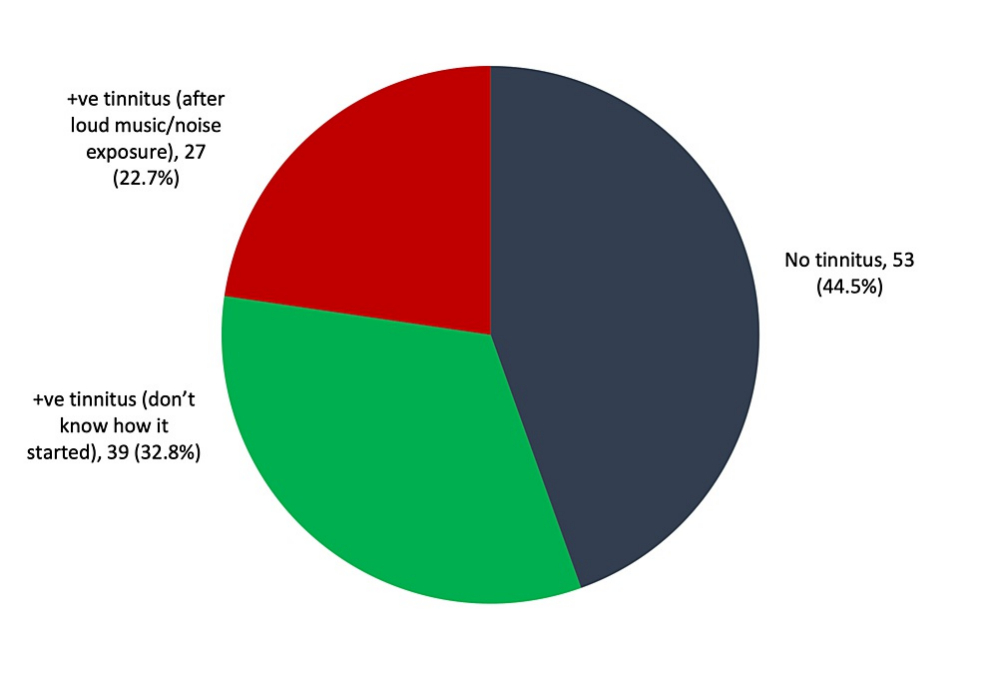
Prevalence of tinnitus among young adults (15-25)

We included 119 young adults aged 15-25 years, with a mean age of 22.5 ± 2.1 years, and 77 (64.7%) were women. Regarding tinnitus prevalence, 66 (55.5%) had tinnitus, out of which 27 (22.7%) had noise-induced tinnitus, 39 (32.8%) had tinnitus of unknown cause, and 53 (44.5%) had no tinnitus (Figure 1). 

In total, 40.3% of females reported having tinnitus, compared to 24.4% of normal females with a calculated significance of (P=.04). Additionally, 38.7% of adults with tinnitus had a bachelor's degree versus 40.3% (P=.005) (Table 1). 

**Table 1:** Bio-demographic data of study groups of adults P: Pearson X2 test $: Exact probability test * P < 0.05 (significant)

Bio-demographic Data		No tinnitus		+ve tinnitus		Total No	P-value
		No	%	No	%		
Age in years	15-20	6	5.0%	13	11.0%	19	.059
	21-25	47	39.5%	53	44.5%	100	
Gender	Male	24	20.2%	18	15.1%	42	.03*
	Female	29	24.4%	48	40.3%	77	
Education level	High school or less	5	4.2%	20	16.8%	25	.004*
	Bachelor and higher	48	40.3%	46	38.7%	94	
Do you use any medications?	Yes	3	2.5%	9	7.6%	12	.150
	No	50	42.0%	57	49.8%	107	
Do you have any allergies?	Yes	9	7.6%	17	14.3%	26	.245
	No	44	36.8%	49	41.2%	93	
Mention allergy type	Antibiotic	1	14.3%	0	0.0%	1	1.0$
	Cats	0	0.0%	1	14.3%	1	
	Dust	2	28.6%	1	14.3%	3	
	Fish	0	0.0%	1	14.3%	1	
	Peanut	0	0.0%	1	14.3%	1	
Medical illness	Asthma	0	0.0%	4	20.0%	4	.773$
	Diabetes	1	5.0%	1	5.0%	2	
	Head trauma	1	5.0%	1	5.0%	2	
	Heart problems	1	5.0%	2	10.0%	3	
	Migraine	0	0.0%	1	5.0%	1	
	Rhinitis unspecified	3	15.0%	5	25.0%	8	

A total of 66.7% of adults with noise-induced tinnitus experienced ringing, roaring, or buzzing in both ears or heads following listening to loud sounds or loud music only. This was compared to 28.2% of adults with unknown causes (P=.002). In total, 33.3% of adults with noise-induced tinnitus had mild/moderate problems caused by ringing, roaring, or buzzing, versus 43.6% of others with tinnitus of unknown cause (P=.141) (Table 2). 

**Table 2:** Noise exposure among adults with tinnitus (noise-induced and unknown) P: Exact probability test * P < 0.05 (significant)

Noise exposure	Group				p-value
	+ve tinnitus (don’t know how it started)		+ve tinnitus (after loud music/noise exposure)		
	No	%	No	%	
In the past month, have you been bothered by ringing, roaring, or buzzing in ears or head that lasts for 5 minutes or more?					.322
Yes	10	25.6%	10	37.0%	
No	29	74.4%	17	63.0%	
If yes, how long have you been bothered?					.287
< 3 months	3	30.0%	1	10.0%	
3 ms-1 year	2	20.0%	3	30.0%	
1-4 years	1	10.0%	4	40.0%	
> 4 years	2	20.0%	0	0.0%	
Don’t know	2	20.0%	2	20.0%	
Have you ever experienced ringing, roaring, or buzzing in your ears/head?					.232
Yes	37	94.9%	27	100.0%	
No	2	5.1%	0	0.0%	
Are you bothered by ringing, roaring, or buzzing in your ears or head only after listening to loud sounds or loud music?					.002*
Yes	11	28.2%	18	66.7%	
No	28	71.8%	9	33.3%	
How much of a problem is this ringing, roaring, or buzzing in your ears or head?					.141
Moderate problem	4	10.3%	5	18.5%	
Minor problem	13	33.3%	4	14.8%	
No problem	19	48.7%	12	44.4%	
Don’t know	3	7.7%	6	22.2%	

Tinnitus was bilateral among 74.1% of adults with noise-induced tinnitus compared to 56.4% of others with unknown tinnitus cause with no statistical significance (P=.340). Moreover, the tinnitus sounds were ringing in 40.7% of the noise-induced group versus 51.3% of the other group, and a pulsating sound in 33.3% and 28.2%, respectively (P=.413). The sound was high among 7.7% of tinnitus with unknown causes compared to none of the noise-induced tinnitus groups (P=.268). Conversely, the sound was continuous in 14.8% of noise-induced tinnitus adults compared to 38.5% of the other group, with a statistically significant difference (P=.037) (Table 3).

**Table 3:** Clinical data of tinnitus among study adults by its type P: Exact probability test * P < 0.05 (significant)

Tinnitus data	Group				p-value
	+ve tinnitus (don’t know how it started)		+ve tinnitus (after loud music/noise exposure)		
	No	%	No	%	
In which ear do you hear tinnitus					.340
Right ear	7	17.9%	3	11.1%	
Left ear	10	25.6%	4	14.8%	
Both ears	22	56.4%	20	74.1%	
What best describes the sound that you hear?					.413
Ringing	20	51.3%	11	40.7%	
Pulsating	11	28.2%	9	33.3%	
Buzzing	6	15.4%	9	33.3%	
Rushing water	5	12.8%	1	3.7%	
Hissing	8	20.5%	6	22.2%	
Crickets	4	10.3%	1	3.7%	
Roaring	1	2.6%	0	0.0%	
On a scale of 0-10, how loud is the sound you hear?					.268
Low (0-4)	21	53.8%	18	66.7%	
Moderate (5-7)	15	38.5%	9	33.3%	
High (8-10)	3	7.7%	0	0.0%	
How do you describe your tinnitus perception?					.037*
Continuous	15	38.5%	4	14.8%	
Intermittent	24	61.5%	23	85.2%	

Of the cases of noise-induced tinnitus, 48.1% were mild, whereas 18.5% were severe versus 33.3% and 28.2% of tinnitus of unknown cause group, respectively, with no statistical significance (P=.444) (Table 4).

**Table 4:** Severity of tinnitus among study adults P: Pearson X2 test TFI: tinnitus functional index

Severity of TFI	Total	Group				p-value
		+ve tinnitus (don’t know how it started)		+ve tinnitus (after loud music/noise exposure)		
	No (%)	No	%	No	%	
Mild	26 (39.4%)	13	33.3%	13	48.1%	.444
Significant	24 (36.4%)	15	38.5%	9	33.3%	
Severe	16 (24.2%)	11	28.2%	5	18.5%	

**Figure 2:**
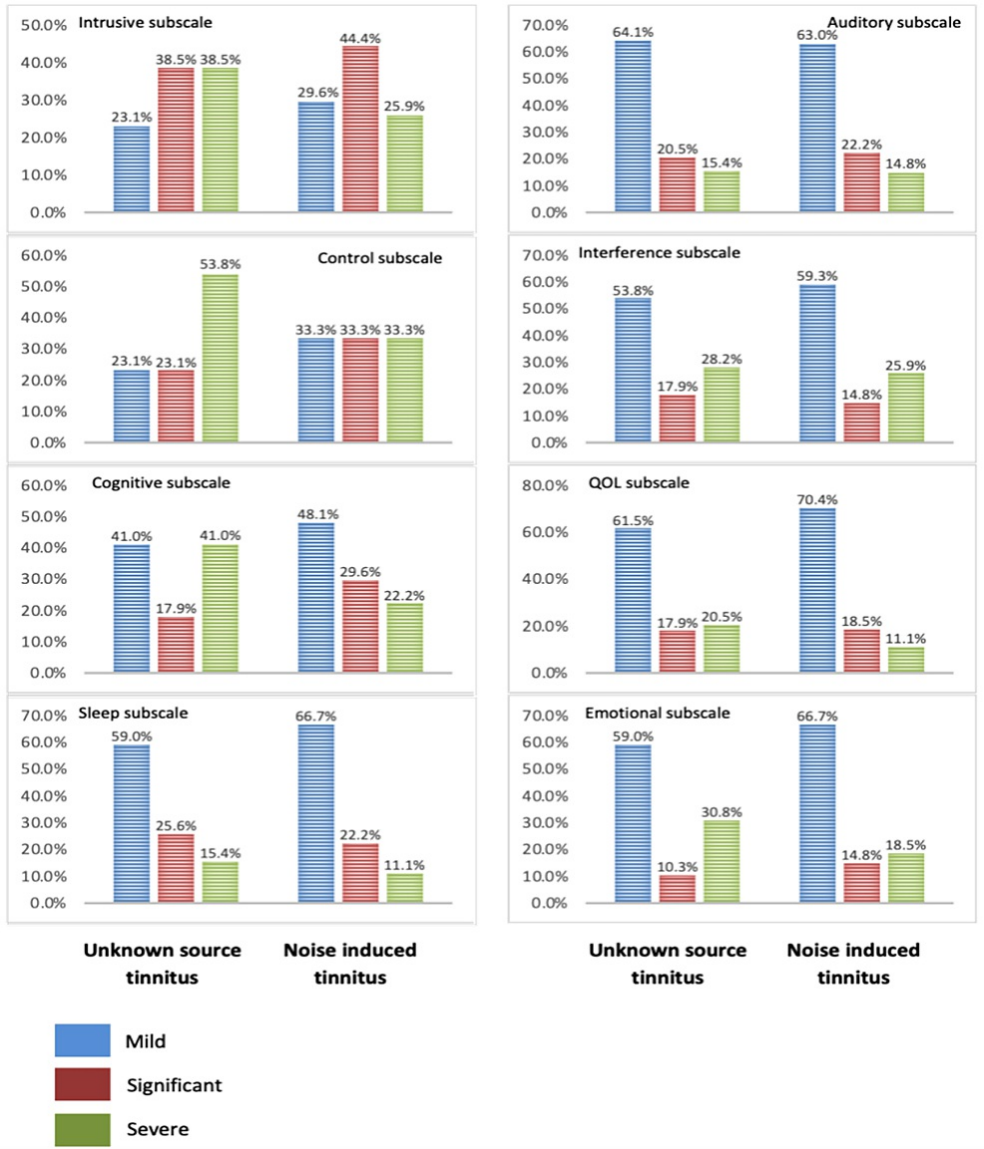
Comparison of tinnitus functional index (TFI) subscales in three categories between noise-induced and unknown source groups

The severity of each subscale was analyzed using the chi-squared test. Insignificant differences were found in all subscales between the two groups, including intrusiveness (χ2=1.1; P=.561), sense (χ2=2.7; P=.258), interference (χ2=2.8; P=.242), sleep (χ2=0.44; P=.801), auditory (χ2=.03; P=.986), relaxation (χ2=.21; P=.901), quality of life (QOL) (χ2=1.1; P=.595), and emotional (χ2=1.4; P=.508) (Figure 2).

## Discussion

Tinnitus is a frequent otological protest reflecting a perception of hearing abnormality, which can be either subjective or objective. Multiple factors can trigger tinnitus, both locally or systemic [13]. Extreme exposure to noise in young adults can result in noise-induced tinnitus. Degeest et al. found a 73% prevalence of transient tinnitus following exposure to excessive leisure noise in young adults [10].

Furthermore, some studies have reported increasing rates of tinnitus among workers in loud environments, including those in the military and dentists while using handpieces and suction [14,15]. One study revealed that the hearing status of young adults was considerably worse for those with an unfavorable attitude toward noise [16]. Among many people with tinnitus, the leading cause remains unidentified and may be triggered by constant exposure to noise [17]. Most cases of tinnitus with identified causes involve malfunctioning of the cochlea, age-related or noise-induced hearing loss, trauma to the head or ear, disorders of the lymphatic system, deficiency in cochlear vascularity, and infections [13].

In this study, 27 (22.7%) young adults stated having tinnitus following the exposure to noise, whereas 39 (32.8%) had tinnitus of unknown cause, accounting for 55.5% of the total sample. These results contradict those found by Musleh et al., where tinnitus accounted for only 28.5% of the sample [18] and an additional study conducted on medical students who owned and used personal sound devices showed that 33% of participants experienced different intensities of tinnitus [19]. The present study identified a significantly high frequency of tinnitus among participants with no history of chronic allergies or health problems. These findings were compatible with those of Musleh et al. and Sunny et al. [18,20]. Furthermore, 66.7% of adults with noise-induced tinnitus were experiencing different perception of sounds. The reported sounds were ringing, roaring, or buzzing of the head or ears following exposure to loud sounds or solitary music. These results conform with those reported by Musleh et al. [18]. In the current study, tinnitus perception was ringing in most of the noise-induced group. This was followed by pulsating sounds and buzzing. Bhatt et al. reported that 73.7%, 21.7%, and 14.6% of patients experience ringing, buzzing, and pulsating sounds, respectively [21].

Substantial evidence indicates a growing risk of music-induced hearing loss in the general population, especially among adolescents and young adults, primarily because listening to loud music lasts for prolonged periods [22]. Another study revealed that many children and teenagers exposed themselves voluntarily to noise through different means, such as headphones, concerts, and car sound systems [23]. In the present study, noise-induced tinnitus was higher than tinnitus of unknown cause in the 15-21 year age group (29.6% and 10.3%, respectively; P=0.048). The severity of noise-induced tinnitus may be related to the level of noise-induced hearing loss (NIHL) [24,25]. In the current study, the majority of participants experienced mild-intensity noise-induced tinnitus in an intermittent pattern (P=0.037). Simultaneously, 18.5% of patients had severe tinnitus, which might have placed them at a higher risk of developing NIHL.

Hence, this highlights the need to improve our understanding and emphasize the complications of hearing loss and its types, which may lead to temporary or permanent hearing impairment. Moreover, this explains the importance of decreasing the volume of sound. Folmer et al. reported a significant positive and measurable impact of a hearing conservation program on the study group [26]. The total TFI scores between the two groups with noise-induced and unknown causes of tinnitus were statistically insignificant. These findings may be due to the inclusion of participants with intact hearing. Moreover, non-objective methods were used to distribute the participants. Mahafza et al. reported a significant variance in the severity of tinnitus assessed using TFI when comparing a group with normal hearing to another group with sensorineural hearing loss [12].

Study limitations

This study was conducted in a single city, on a specific age group and limited population. National studies including multiple age groups from various backgrounds with larger numbers are recommended.

## Conclusions

Tinnitus is a common complaint with no definite pathophysiology yet identified. High prevalence of tinnituswas found in this study, and it was suggested by the literature as well. Several triggers are purportedly related to the development of tinnitus. Constant exposure to loud noise is considered a significant risk factor for tinnitus. Young adults require proper education about the causes of tinnitus and other hearing abnormalities and more importantly, methods to protect and maintain their ear health.
